# Loop‐Mediated Isothermal Amplification (LAMP) for the Diagnosis of Sexually Transmitted Infections: A Review

**DOI:** 10.1111/1751-7915.70153

**Published:** 2025-05-02

**Authors:** Yasaman Ahmadi, Yejiong Yu, Zhanfeng Cui, Wei E. Huang, Monique I. Andersson

**Affiliations:** ^1^ Department of Engineering Science University of Oxford Oxford UK; ^2^ Department of Microbiology Oxford University Hospitals NHS Foundation Trust Oxford UK; ^3^ Nuffield Division of Clinical Laboratory Science, Radcliffe Department of Medicine University of Oxford Oxford UK

**Keywords:** *Chlamydia trachomatis*, hepatitis B virus, herpes simplex virus, human immunodeficiency virus (HIV), loop‐mediated isothermal amplification (LAMP), *Neisseria gonorrhoeae*, point‐of‐care tests (POCT), sexually transmitted infections (STIs), *T. pallidum* subspecies pallidum, *Trichomonas vaginalis*

## Abstract

Sexually transmitted infections (STIs) remain a significant public health concern. Given the asymptomatic nature of many STIs, diagnostic testing is critical for determining the appropriate treatment, enabling effective tracing and reducing the risk of further transmission. Nucleic acid amplification tests (NAATs) are the most sensitive and the most widely used in well‐resourced settings. The majority of available NAATs are based on polymerase chain reaction (PCR), which requires highly trained personnel and costly equipment, making it impractical for resource‐limited settings. Loop‐mediated isothermal amplification (LAMP) has emerged as a simple, rapid, sensitive and low‐cost alternative for pathogen detection, particularly well‐suited for point‐of‐care tests (POCT). In this review, we evaluate LAMP assays reported in the literature for the detection of pathogens linked to the high incidence STIs prioritised by the World Health Organization (WHO) for POCT in 2023. These include *Neisseria gonorrhoeae* , *Chlamydia trachomatis* , *Trichomonas vaginalis*, *T. pallidum* subspecies *pallidum*, as well as other common STIs such as herpes simplex virus, hepatitis B virus and human immunodeficiency virus (HIV). For each LAMP assay, we identified and summarised the key elements such as the type and number of tested clinical specimens, chosen target gene, detection system, reference test and clinical outcomes. We highlight the advantages and limitations of these assays and discuss the gaps that should be addressed to improve their applicability for POCT.

## Graphical abstract

LAMP assay for point‐of‐care tests.

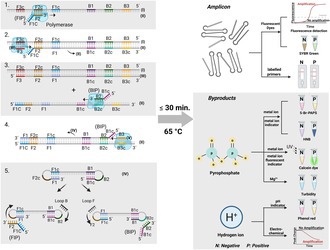

## 1 Introduction

Sexually transmitted infections (STIs) are a major public health problem. *Neisseria gonorrhoeae* , *Chlamydia trachomatis* and *T. pallidum* subspecies *pallidum* are the three curable, most reported bacterial causes of STIs worldwide. Trichomoniasis is another common STI caused by the protozoan *Trichomonas vaginalis*. World Health Organization (WHO) data suggest that there are 357 million new cases of these four STIs per year among people aged 15–49 years (WHO 2021; https://apps.who.int/iris/handle/106). Herpes simplex virus (HSV), hepatitis B virus (HBV) and human immunodeficiency virus (HIV) are among the most prevalent viral STIs. HIV has caused > 40 million deaths as of 2021, and HBV annually causes 780,000 deaths/year (Jefferies et al. 2018). These infections cause a significant burden of disease (GBD 2015 Disease and Injury Incidence and Prevalence Collaborators 2016) and disproportionately affect women and children (Beck Sagué et al. 2014; Van Gerwen et al. 2022). These figures contrast sharply with the importance that these infections are given in terms of clinical and public health practice and research.

In many under‐resourced settings, where the burden is the highest, syndromic treatment of STIs is the standard management. This strategy results in missed opportunities for treatment, which can lead to the administration of the wrong treatment or overtreatment, the latter contributing to the spread of antimicrobial resistance. Even in well‐resourced areas, symptomatic patients are often treated empirically because of the delay in the availability of results from laboratory‐based testing.

Historically, several different laboratory methods have been available for detecting STIs such as culture, microscopy, antigen detection, antibody serology testing and nucleic acid amplification tests (NAATs). Comprehensive reviews of these methods have been previously published (Tang and Ou 2012; Muralidhar 2015; Edwards et al. 2016; Meyer 2016; Luo et al. 2020; Meyer and Buder 2020; Poljak et al. 2020; Caruso et al. 2021; Lee and Kim 2021; Nath et al. 2021). Culture is rarely used for the diagnosis of STIs, aside from its application to determine and monitor antibiotic susceptibility in *N. gonorrhoeae* (Papp et al. 2014). Microscopic examination of vaginocervical scrapes by ‘wet mount’ allows rapid and affordable diagnosis of trichomoniasis and is the method of choice in low‐resource settings. However, this test has considerably lower clinical sensitivity compared to NAATs to detect *T. vaginalis* (Patil et al. 2012). The immunoassays based on detecting the pathogen antigen or antibody can provide a quick and cost‐effective method for STI diagnostics. However, such rapid tests do not perform adequately or are currently not available for all STIs. For example, the detection of *N. gonorrhoeae* and *C. trachomatis* antigens or antibodies by immunoassays is not recommended for clinical testing due to insufficient sensitivity and specificity of the available tests (Papp et al. 2014). NAATs are currently the gold standard for detecting *N. gonorrhoeae* (Meyer and Buder 2020) and *C. trachomatis* (Meyer 2016) and serve as the primary diagnostic test for detection and subtyping HSV in swab samples from skin, oral and genital lesions (Nath et al. 2021). The gold standard for the diagnosis of *T. vaginalis* in high‐resource settings is NAATs (Edwards et al. 2016). NAATs are used for the follow‐up of patients infected with HIV or HBV to monitor viral loads and response to antiviral therapy (Iloeje et al. 2006; Drain et al. 2019). Detecting *T. pallidum* in mucosa and skin lesions of suspected primary and secondary syphilis by NAATs is a sensitive method. NAAT is a particularly powerful tool for the diagnosis of primary syphilis (Luo et al. 2020), particularly where a serological response has yet to develop (Heymans et al. 2010; Shields et al. 2012).

Many NAATs, mostly based on real‐time PCR, have been developed for the diagnosis of STIs and are commercially available. PCR assays, however, need highly trained staff and sophisticated costly instrumentation, precluding their use in resource‐limited countries where the burden of these infections is highest (Peters et al. 2022). Besides, many PCR assays have a time‐to‐result of several hours, necessitating a second visit of patients to the clinic, causing loss to follow‐up and reduced opportunity for contact tracing (Rolland et al. 2020).

Point‐of‐care (PoC) testing, compliant with the ASSURED (Affordable, Sensitive, Specific, User‐friendly, Rapid, Equipment free and Delivered to users) criteria developed by the WHO (Peeling et al. 2006), would provide a powerful tool to reduce the incidence of STIs (Hsieh et al. 2011; Drancourt et al. 2016; Toskin et al. 2017; Unemo et al. 2017). The importance of PoC tests for the diagnosis of STIs has been reviewed previously (Tucker et al. 2013; Gaydos and Hardick 2014; Unemo 2021; Vargas et al. 2022). These reviews indicate the rising interest in isothermal amplification methods as prime candidates for use in STI PoC diagnostics as they can be simple, rapid, inexpensive and require little equipment. Several isothermal amplification methods are available including LAMP (loop‐mediated isothermal amplification), RPA (recombinase polymerase amplification), HAD (helicase‐dependent amplification) and NASBA (nucleic acid sequence‐based amplification) among others. In Table 1, these technologies are briefly described, and their respective advantages and disadvantages are highlighted. For a comprehensive description of variant isothermal amplification assays and their comparison, the reader is referred to recent reviews by (Glökler et al. 2021; Oliveira et al. 2021).

**TABLE 1:** Comparison of various isothermal amplification methods.

Method	LAMP	RPA	HDA	NASBA
Principle	4–6 primers bind to 6–8 distinct regions of target DNA; amplification by strand displacing polymerase	Recombinase binds primers to target DNA in the presence of SSB; amplification by strand displacing polymerase	Helicase unwinds DNA in the presence of SSB; amplification by strand‐displacing polymerase	First primer binds to RNA template, reverse transcriptase (RT) produces RNA–DNA hybrid; RNAse H degrades RNA and RT synthesizes dsDNA, which is then transcribed to RNA by T7 RNA polymerase
Number of primers	4–6	2	2	2
Number of enzymes	1	2	2	3
Operating temperature	60°C–65°C	37°C–42°C	60°C–65°C	41°C
Advantages	Low‐cost, simple set‐up, rapid (< 30 min), highly specific, versatile detection methods	Rapid (15–30 min), low operating temperature, PCR primers may be used, multiplexing capibility	High fidelity in DNA replication	RNA‐specific, free from DNA interference
Disadvantages	Complex primer design, limited multiplexing capability, more prone to amplicon contamination than others	High reagent costs, non‐specific amplification risk	Complicated buffer optimization, slow (1–2 h), less robust than others	Risk of RNA degradation, not suitable for DNA detection, slow (1.5–2 h)

Abbreviations: HAD, helicase‐dependent amplification; LAMP, loop‐mediated isothermal amplification; NASBA, nucleic acid sequence‐based amplification; RPA, recombinase polymerase amplification; SSB, single‐stranded DNA‐binding proteins.

**FIGURE 1:**
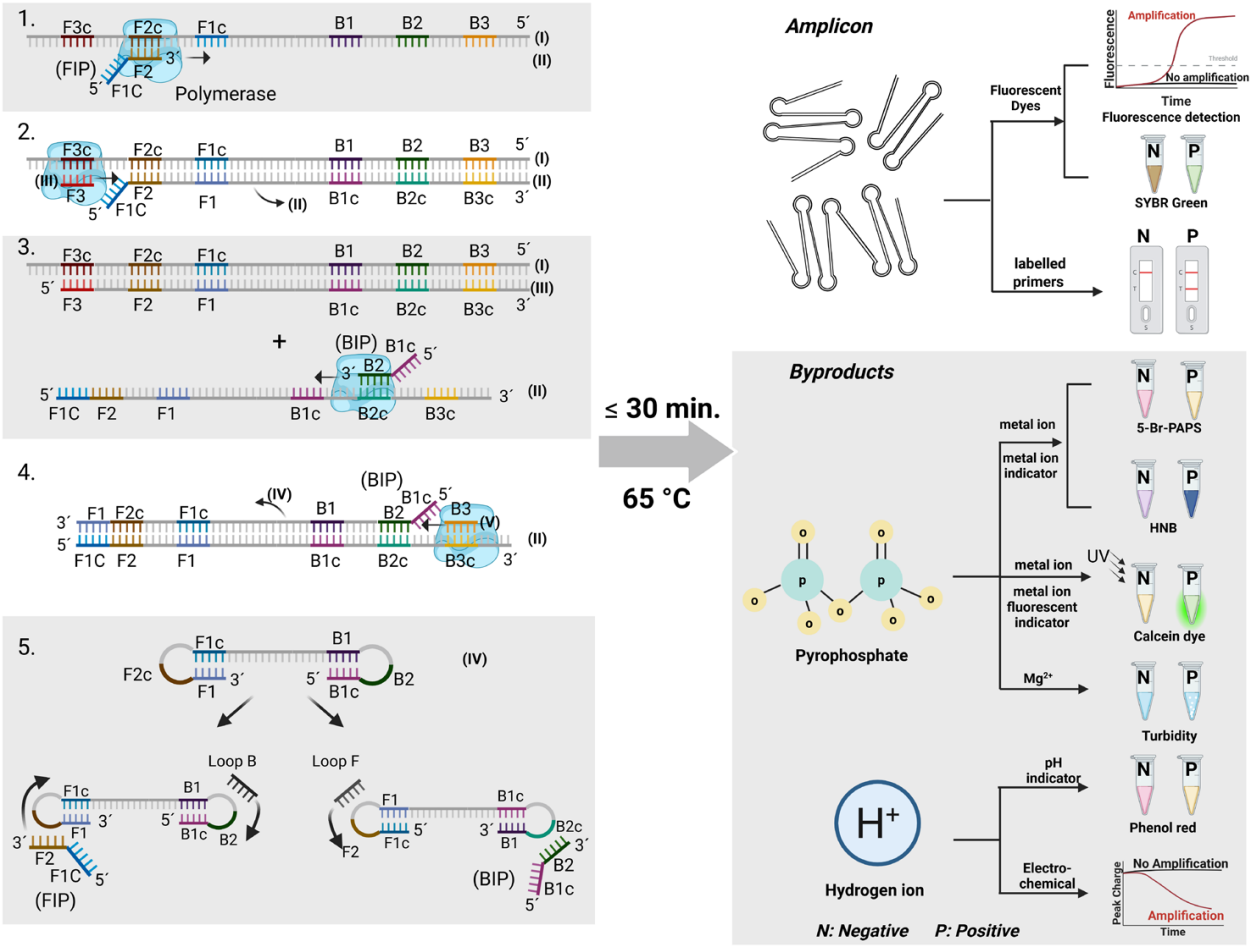
Schematic representation of the mechanism of LAMP (left) and common detection methods (right). The left figure shows the process that starts from primer FIP, however, DNA synthesis can also begin from primer BIP. The formation of a dumbbell‐shaped structure (iv) acts as a seed for exponential amplification for generation of amplicons of various sizes. As shown in the right figure, different detection methods can be applied for results readout as indicated by amplicon and by‐products. For amplicon detection, the most common method is to include intercalating fluorescent dyes such as SYBER Green or Syto9 in the LAMP reaction; amplification can be monitored by real‐time/end‐point fluorescence or alternatively detected visually under UV or blue light. Visual fluorescence detection under visible light is also possible, however, the colour change (yellow/orange to light green) is subtle. With labelled primers, amplicons can be also detected on a lateral flow detection (LFD) strip. Furthermore, some detection methods target by‐products namely pyrophosphate and hydrogen ions. Pyrophosphate precipitates with certain metal ions such as Mg^2+^, Zn^2+^ and Mn^2+^; the metal ion depletion can be further detected using colorimetric metal indicators such as hydroxy naphthol blue (HNB) (Goto et al. 2009) and pyridylazophenol dyes (PAPS) (Zhang, Hunt, et al. 2022; Szobi et al. 2023) or alternatively by using fluorescent metal ion indicators such as calcein (Tomita et al. 2008). Additionally, the precipitation of magnesium pyrophosphate results in a change in the turbidity of the solution, which can be detected by a turbidimeter (Mori et al. 2001). For hydrogen ion detection, a colorimetric pH indicator such as phenol red (Tanner et al. 2015) can be incorporated in the reaction. In addition, the release of hydrogen ions can be detected by electrochemical measurements (Wang et al. 2024).

LAMP is one of the most rapid isothermal nucleic acid amplification techniques (Notomi et al. 2000). The benefits and advancements in LAMP diagnosis of diseases have been reviewed recently (Moehling et al. 2021; Das et al. 2022). LAMP uses four main primers including two outer primers (F3 and B3) and two inner primers (FIP and BIP) designed to target six‐specific regions within the template sequence. The addition of two loop primers (LF and LB) further accelerates amplification by reducing the reaction time to less than half that of the original LAMP (Nagamine et al. 2002). LAMP with six primers can be accomplished in ≤ 30 min at a single temperature of 63°C–65°C. Quantitative analysis in LAMP can be obtained by measuring time‐to‐threshold (TT), a value similar to cycle threshold (Ct) in real‐time PCR (Yu et al. 2022). The yield of amplification in LAMP is 50–100 times more than PCR (10–20 μg amplified DNA in LAMP vs. 0.2 μg in PCR) (Mori et al. 2001). The high amplification yield in LAMP and thus large formation of by‐products, namely pyrophosphate ions and hydrogen ions, allows versatile detection mechanisms for results readout. The mechanism of LAMP and common detection methods are illustrated in Figure 1.

LAMP assays for STIs could fulfil the ASSURED criteria and would be a viable option for PoC use. The aim of this article is to provide an overview of STI LAMP assays reported in the literature, which have been validated using clinical samples. We summarised the features and clinical results of these assays, highlighted their advantages and limitations, and further discussed the requirements that still need to be met to enhance the PoC adaptability of LAMP assays.

## 2 Methods

**FIGURE 2:**
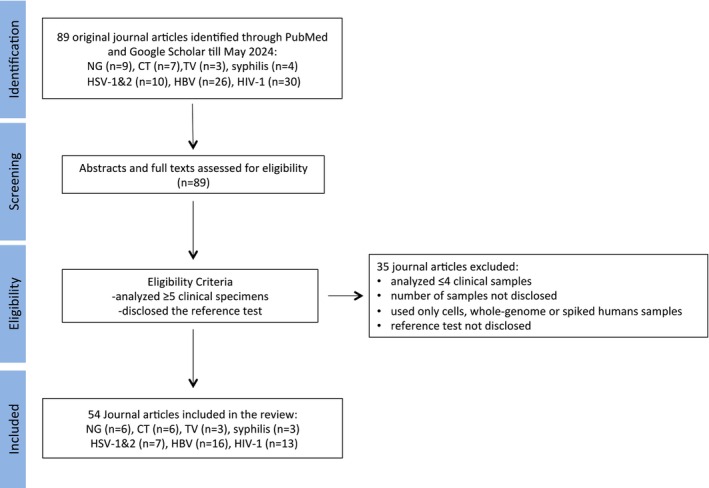
Flow diagram detailing the selection and review process. CT, *Chlamydia trachomatis* ; HBV, hepatitis B virus; HIV, human immunodeficiency virus; HSV, herpes simplex virus; NG, *Neisseria gonorrhoeae* ; Syphilis, *T. pallidum* subspecies *pallidum*; TV, *Trichomonas vaginalis*.

We considered peer‐reviewed original journal articles published till March 2024. PubMed and Google Scholar were sources of the literature. Articles that reported developing or using the LAMP technique for the diagnosis of *Neisseria gonorrhoeae* (NG), *Chlamydia trachomatis* (CT), *Trichomonas vaginalis* (TV), *T. pallidum* subspecies *pallidum*, HSV‐1/2, HBV and HIV‐1 were evaluated. Only those studies that tested five or more (≥ 5) clinical samples were included in this review. We excluded studies that evaluated only four or fewer clinical samples or only used spiked human specimens, cells or whole genome for validating their assays. We included only journal articles that disclosed the number of tested clinical samples and their reference test. The full texts of all potentially eligible articles were reviewed. Our search yielded 89 publications. We excluded 35 journal articles, resulting in 54 journal articles, which are classified in Tables 2, 3, 4, 5, 6, 7, 8. Figure 2 depicts the flow diagram of searched journal articles. We stratified the results for the target gene, type and number of clinical specimens, the choice of sample pre‐treatment, the chosen detection platform (gel electrophoresis (GE), real‐time fluorescence, calcein detection, colorimetric and turbidity among others) and the selected reference test (culture, microscopy, immunoassay, real‐time PCR, real‐time quantitative PCR (qPCR) or conventional PCR with GE). The manufacturer of reference tests and extraction kits was noted if provided in the publication. In addition, a brief clinical outcome of each reported assay is included in the tables.

## 3 Results

### 3.1 *Neisseria gonorrhoeae* (NG)

We identified six papers for detecting NG by LAMP (Table 2).

**TABLE 2:** LAMP for detection of *Neisseria gonorrhoeae* (NG) and resistance.

Study	Target gene	Sample	Sample pre‐treatment	Reference tests	Detection mechanism	Results summary
Xu et al. (2016)	*cppB*	6 swabs	Magnetic bead extraction in a capillary platform	Real‐time PCR	Calcein detection	3 negative and 3 positive both in LAMP and PCR Multiplex NG/CT detection in a capillary system
Liu et al. (2017)	*porA*	26 genital swabs	DNA extraction by a commercial kit	Culture	Visual colour detection of Syto9	18 samples positive by both culture and LAMP. One sample positive only by culture and one sample only positive by LAMP, which regarded as TP and FP, respectively
Shimuta et al. (2019)	*penA* & *penA‐60.001*	12 urethritis swabs	Heating & centrifugation^a^	Culture	Not indicated	7 NG‐positive and 5 NG‐negative samples by both culture and LAMP; no resistance detected
Chen, Zhou, et al. (2021)	*orf1*	86 genital swabs	Crude lysis by nucleic acid–releasing agents	Culture & real‐time PCR (DaAn Gene Co)	LFD	58 samples were positive by both culture and LAMP, only 55 of them were positive by PCR. All culture‐negative samples were negative in LAMP and PCR
Shimuta et al. (2022)	*pen A* & *non‐mosaic penA*	101 urethral swabs	DNA extraction (QIAmp DNA minkit)	Culture, Roche Cobas real‐time PCR, sequencing	Calcein detection	6 negative and 95 positive samples for NG by culture, PCR and *penA*‐LAMP. Of 95 *penA*‐positive samples, both sequencing and mosaic *penA*‐LAMP identified 39 positive (susceptible NG) and 56 negative samples (resistant NG)
Chen et al. (2023)	*orf1*	146 vaginal swabs	Crude lysis by nucleic acid–releasing agents	qPCR (DaAn Gene Co)	LFD & colorimetric with Malachite green	Full agreement between qPCR and LAMP, both identified 30 NG‐positive samples Multiplex NG/CT detection

Abbreviations: FN, false‐negative; FP, false‐positive; LFD, lateral flow detection; qPCR, quantitative PCR.

^a^ Clinical specimens were heated at 95°C for 5 min, followed by centrifugation at 9000 *g* to pellet the cells. The supernatant was used for LAMP or PCR assay.

Xu et al. developed a capillary‐based magnetically actuated platform for multiplex detection of NG and CT (Xu et al. 2016). The focus of this work has been on the platform and only six clinical swabs (swab type was not indicated) were analysed, which showed consistent data with real‐time PCR. This integrated capillary system is comprised of different parts including a lysis buffer, magnetic beads to capture DNA, washing buffer to remove inhibitors prior to the amplification and a LAMP reaction mixture among others. By moving a handheld magnet, the DNA is passed through a series of distinct process steps enabling purification, enrichment and amplification of the sample. The results could be read by the naked eye through illuminating the device under UV flashlight.

Liu et al. designed LAMP primers targeting the *porA* pseudogene and validated their assay on 26 genital samples, and used culture as the reference test (Liu et al. 2017). Both culture and LAMP identified 18 positive samples. One sample was positive only by culture, and one sample was positive only in LAMP, which the authors considered as true‐positive and false‐positive, respectively, resulting in the clinical sensitivity of 95% (18/19) and specificity of 86% (6/7) for LAMP.

Chen et al. developed a LAMP assay targeting the *orf1* gene and analysed 86 genital swabs for validation (Chen, Zhou, et al. 2021). Cultivation was used as the reference test. Here, a simple method was used for sample pretreatment by adding nucleic acid–releasing agents to specimens in a 1:1 stoichiometric ratio followed by incubation at room temperature for 10 min. Both culture and LAMP identified 28 samples as negative and 58 samples as positive, leading to clinical sensitivity and specificity of 100% for LAMP. Real‐time PCR by a commercial kit (DaAn Gene Co. Ltd., China) detected only 55 of 58 (95%) positive samples.

Two studies developed LAMP assays for detecting the antibiotic resistance genes in *N. gonorrhoeae* . Successive development of resistance to penicillin, tetracycline and fluoroquinolones has limited the therapeutic use of these antibiotics for treatment of NG infection (Unemo and Shafer 2011; Wi et al. 2017). Following WHO recommendations (2021), most countries are currently using a monotherapy with ceftriaxone (Derbie et al. 2020; Kueakulpattana et al. 2021). The only commercially available molecular technology to address *N. gonorrhoeae* resistance is the ResistancePlus GC (Roche & SpeeDx), which is a multiplex (five channels) real‐time PCR test to detect NG and sequences linked to ciprofloxacin susceptibility. The mechanism of NG resistance to ceftriaxone and azithromycin is mediated through multiple mechanisms, with mutations in the mosaic *penA* gene (causing mutations in penicillin‐binding protein) as one of the major mechanisms. In this regard, Shimuta et al. (2019) developed two LAMP assays by targeting regions of the *penA gene* (for detecting NG) and the *penA*‐60.001 gene (for detecting ceftriaxone‐resistant NG). The specimens were heat‐inactivated, followed by centrifugation and collecting the supernatant for LAMP. The specimens were considered to contain resistant NG only if both LAMP assays gave positive results. The authors reported successful detection of resistant strains among 299 bacterial strains, including 204 *N. gonorrhoeae* strains and 95 strains of other *Neisseria* species. Furthermore, 12 urethritis specimens were analysed; seven of them were confirmed positive by both LAMP and culture; however, none was resistant. In another work by the same group (Shimuta et al. 2022), two LAMP assays were developed for detecting cefixime‐susceptible *N. gonorrhoeae* . Two primer sets were designed, one targeting the *penA* gene (to detect NG) and one for targeting the non‐mosaic *penA* gene (to detect cefixime‐susceptible NG). A total of 101 urethral swabs of male patients with urethritis were tested, of which 95 were confirmed to be NG positive by culture, real‐time PCR and *penA*‐LAMP. Within positive samples (*n* = 95), 39 of them were detected to be positive for non‐mosaic *penA*‐LAMP (susceptible NG), while 56 were negative (resistant NG). PCR followed by Sanger sequencing confirmed the LAMP results.

In a study by Chen et al., a multiplex LAMP assay was developed for the detection of NG and CT in a single tube reaction using fluorescently labelled primers (Chen et al. 2023). Primers against the *orf1* gene of *N. gonorrhoeae* were designed. LAMP was in full agreement with quantitative PCR (qPCR). Of 146 genital swabs, qPCR (DaAn Gene Co. Ltd., China) and LAMP confirmed 30 samples as NG‐positive, 29 of them including > 500 copies per test and one sample with 100–500 copies. The latter was additionally confirmed to be true‐positive using PCR‐sequencing. All qPCR‐negative samples were also negative in LAMP.

### 3.2 *Chlamydia trachomatis* (CT)

For detecting CT by LAMP, we identified six studies (Table 3).

**TABLE 3:** LAMP for detection of *Chlamydia trachomatis* (CT).

Study	Target gene	Sample	Sample pre‐treatment	Reference test(s)	Detection mechanism	Results summary
Xu et al. (2016)	Cryptic plasmid	6 swabs	Magnetic bead extraction in a capillary platform	Real‐time PCR	Calcein detection	Full agreement between LAMP and PCR: 4 negative and 2 positive CT samples. Multiplex NG/CT detection in a capillary system
Choopara et al. (2017)	*ompA* ^a^	284 endocervical swabs	Crude cell lysis^b^	PCR‐GE & sequencing	GE & colorimetric with HNB	Within 31 pre‐identified positive samples, 27 and 26 samples tested positive by PCR and LAMP, respectively. LAMP led to 11 undetermined samples due to nebulous colour of HNB
Somboonna et al. (2018)^c^	*ompA* ^a^	130 endocervical swabs	Crude cell lysis^b^	PCR‐GE & sequencing	GE & colorimetric with AuNP	Within 25 pre‐identified positive samples, PCR could detect all of them (100% sensitivity), while LAMP detected 23 of them (92% sensitivity). PCR also led to 2 FP cases. LAMP specificity: 100%
Chen et al. (2022)	*ompA* ^a^	87 genital secretion samples	Centrifugation & cell lysis	qPCR	Real‐time turbidity, colorimetric with Malachite green, LFD	Full agreement between LAMP and qPCR: 43 positive samples, 44 negative samples
Dean et al. (2021)	rRNA	353 vaginal swabs	Crude cell lysis by a proprietary buffer	Cepheid Xpert CT/NG PCR assay & qPCR for discrepant samples	Colorimetric	63 positive samples by Xpert test; 59 of those also positive in LAMP. Among 290 Xpert‐negative samples, 288 were LAMP‐negative. Discrepant samples (*n* = 7) resolved to be 4 FN, 2 FP and one TP for LAMP
Chen et al. (2023)	*ompA* ^a^	146 vaginal swabs	Crude lysis by nucleic acid–releasing agents	qPCR (DaAn Gene Co)	Multiplex LFD & Colorimetric with Malachite green	Full agreement between qPCR and LAMP, both identified 51 CT‐positive samples Multiplex NG/CT detection

Abbreviations: FN, false‐negative; FP, false‐positive; GE, gel electrophoresis; LFD, lateral flow detection; qPCR, quantitative PCR; TP, true‐positive.

^a^ Degenerate primers.

^b^ Mixing 40 μL of the endocervical swab sample (in M4RT buffer) with 20 μL of tris‐EDTA buffer, followed by heating at 95°C for 5 min.

^c^ Same primers, sample pre‐treatment and reference test as Choopara et al. (2017).

Choopara et al. designed degenerate primers for conserved regions of *ompA* sequences of 19 known CT serological variants including serovars A–K, Ba, Da, Ia, Ja, L1, L2, L2a and L3 (Choopara et al. 2017). They verified their assay on 284 endocervical swab specimens from clinically symptomatic patients (*n* = 150) and healthy individuals (*n* = 134). For sample pre‐treatment, a simple crude DNA lysis step was used by mixing the swab sample with Tris‐EDTA buffer and heating. Among 31 pre‐identified positive samples (not mentioned by which assay), 27 and 26 samples were tested positive by PCR targeting the *ompA* gene and LAMP, respectively. Within 284 specimens, three samples were positive only by PCR and LAMP, which were regarded as false‐positives. LAMP, however, resulted in 19 undetermined samples (out of 284) due to nebulous colour change of HNB dye. To address this issue, in another work by the same group (Somboonna et al. 2018), a colorimetric readout based on gold nanoparticle (AuNP) was developed. Here, an AuNP‐DNA probe specific for the LAMP product was prepared and added to the completed amplification reaction. The reaction was further incubated at 61°C to allow hybridization of the LAMP product to its complementary DNA sequence on the AuNP, followed by adding salt (NaCl or MgSO_4_). The reaction was identified as positive via a purple/blue/grey (negative) to red (positive) colour change, monitored by UV–Vis spectrophotometry or the naked eye. Here, no indeterminate sample was reported. However, such a detection approach needs extra time for hybridization and requires opening the reaction vials to add the AuNP and salt, introducing the risk of amplicon contamination. Based on the represented data, the colour change remained vague, despite these modifications.

Chen et al. designed degenerate primers for the *ompA* gene of 14 known CT serological variants including serovars A‐K, L1, L2 and L3 (Chen et al. 2022). A simple pre‐treatment step was applied by centrifuging (12,000 *g*, 5 min) vaginal swabs to pellet the bacterial cells followed by suspending the pellet in nucleic acid‐releasing agents, allowing the cell debris to settle and further using the supernatant for the LAMP assay. Among 87 genital swabs, 43 samples were confirmed to be positive by both LAMP and qPCR. All PCR‐negative samples were negative in LAMP, leading to 100% clinical sensitivity and specificity for LAMP.

Dean et al. developed a LAMP assay using primers targeting the rRNA gene. The assay was verified on 353 vaginal swabs from 327 females (Dean et al. 2021). The swabs were pre‐treated with a proprietary lysis buffer. The reference Cepheid Xpert CT real‐time PCR identified 63 positive samples; 59 of those were also positive in LAMP (94% sensitivity). Among 290 negative samples identified by the Xpert assay, 288 were also LAMP‐negative. One sample was only positive in LAMP. The discrepant samples (*n* = 7) were further purified using the QIAamp DNA extraction kit and tested by a qPCR targeting the *ompA* gene, which resolved to be four false‐negatives, two false‐positives and one true‐positive for LAMP. Compared to the Xpert assay, LAMP had clinical sensitivity and specificity of 94% and 99.3%, respectively, after discrepant resolution, with an overall accuracy of 98%.

Chen et al. developed a multiplex LAMP assay for detecting CT and NG in a single tube reaction (Chen et al. 2023). For CT detection, degenerate primers against the *ompA* gene of 14 known CT serological variants (A‐K, L1‐L3) were designed. Among 146 genital swabs, 51 of them were confirmed as CT positive by both qPCR (DaAn Gene Co. Ltd., China) and LAMP; 49 samples had > 500 copies per test and three samples had 100–500 copies. The latter samples were additionally confirmed to be true‐positives using PCR‐sequencing. All qPCR‐negative samples were also negative in LAMP. Compared to qPCR, LAMP showed 100% clinical sensitivity and specificity.

### 3.3 Trichomonas vaginalis

For TV diagnosis by LAMP, we identified three papers (Table 4).

**TABLE 4:** LAMP for detection of *Trichomonas vaginalis* (TV).

Study	Target gene	Sample	Sample pre‐treatment	Reference test(s)	Detection mechanism	Results summary
Goo et al. (2016)	Actin type 6	50 vaginal swabs	DNA extraction (QIAamp DNA mini kit) & heat‐treatment	Wet mount microscopy, PCR & multiplex PCR	GE, turbidity & Calcein detection	12 positive samples by microscopy, 23 by multiplex PCR, 40 by PCR. LAMP was positive for 43 samples including those detected by PCR. Similar LAMP results were observed for DNA extracted and heat‐treated samples
Adao and Rivera (2016)	18S rRNA	121 vaginal swabs	Chelex‐100 DNA extraction	Culture & PCR‐GE	GE & visual colour detection of SYBER Safe^a^	53 (44%) samples positive in LAMP, 10 (8%) positive in culture and 9 (74%) in PCR. Discrepant samples (*n* = 44) were not resolved
Khurana et al. (2020)	18S rRNA^b^	153 vaginal swabs	Chelex‐100 DNA extraction	Wet microscopy, PCR‐GE & Cepheid Xpert real‐time PCR assay	Real‐time fluorescence	l28 microscopy‐positive samples were also positive by all three NAATs (PCR, Xpert & LAMP). Of 125 microscopy‐ negative samples, 121 and 3 samples were negative and positive, respectively by all NAATS; LAMP had one additional positive sample

Abbreviations: FP, false‐positive; GE, gel electrophoresis.

^a^ The reaction vials were opened by the end of the reaction to add SYBER Safe.

^b^ Same primer sequences as Adao and Rivera (2016).

Goo et al. verified their assay on 50 vaginal swabs from female patients with clinical suspicion of trichomoniasis (Goo et al. 2016). They designed primers specific to several candidate genes, such as actin, *Tvk* and *β‐tubulin*. Only primers against the actin gene led to amplification. Of 50 vaginal samples, 12 (24%), 23 (46%), 40 (80%) and 43 (86%) were determined to be *T. vaginalis‐*positive using wet mount microscopy, multiplex PCR, PCR targeting the *tvk* gene and LAMP, respectively. All 40 positive specimens identified by PCR were also positive in the LAMP assay. No further verification was conducted for the three samples that were positive only in LAMP. The results of the LAMP assay using heat‐treated samples were consistent with those using purified DNA extracted from samples by a commercial kit.

Adao and Rivera developed LAMP targeting the 18S rRNA gene (Adao and Rivera 2016). Among 121 vaginal swabs, 53 of them (44%) were positive in LAMP, 9 (7%) in conventional PCR‐GE and 10 (8%) by culture. Such a large discrepancy in the performance of LAMP and PCR has not been reported in any other works we reviewed. The authors conducted no further investigation to resolve the source of such a large disagreement between LAMP and PCR. As LAMP is more prone to amplicon contamination than other NAATs (50–100 times more amplification yield than in PCR), we suspect that opening reaction tubes after amplification to add SYBR Safe for UV or GE visualisation (as stated in the original article) may have led to contamination error. It is therefore unclear how many of the discrepant results may have been false‐positive.

Using the same set of primers as (Adao and Rivera 2016), Khurana et al. further developed a LAMP assay for detecting TV in vaginal swabs among symptomatic women (Khurana et al. 2020). All samples were additionally analysed with wet mount microscopy, conventional PCR‐GE targeting the 18S rRNA gene and Cepheid Xpert TV real‐time PCR assay. The authors considered a composite reference standard as the gold standard. Infection with TV was defined as positive if any of the following criteria were met: the presence of motile TV on wet‐mount microscopy and/or any two positive molecular tests (PCR‐GE, Xpert PCR or LAMP). A total of 153 patients were examined. Within the 28 microscopy‐positive cases, all were positive in the three NAATs. Among 125 microscopy‐negative cases, 121 of them were identified as negative by all three NAATs. Three samples among 125 microscopy‐negative samples were detected as positive by all three NAAT. One sample was positive only by LAMP, which was regarded as a false‐positive. The clinical sensitivity of all NAAT assays was 100% and the clinical specificity was 100%, 100% and 99% for PCR, Xpert PCR and LAMP, respectively.

### 3.4 *T. pallidum* Subspecies *pallidum*

We identified three papers for detecting *T. pallidum* subspecies *pallidum by* LAMP (Table 5).

**TABLE 5:** LAMP for detecting *Treponema pallidum* subspecies *palladium* (Syphilis).

Study	Target gene	Sample	Sample pre‐treatment	Reference test(s)	Detection mechanism	Results summary
Xiao et al. (2017)	*bmp*	722 blood samples	DNA extraction (Wizard Genomic DNA kit)	TPPA & PCR	GE, UV detection of SYBER Green	603 TPPA‐positive samples, 493 (82%) and 262 (43%) of those also positive in LAMP and PCR, respectively. 129 TPPA‐negative; 109 and 111 of those negative by LAMP and PCR, respectively
Tarumoto et al. (2020)	23S rRNA	81 skin swab samples	95°C, 5 min	PCR & sequencing	Real‐time turbidity	PCR: 66 positive (11 WT, 55 MT) and 15 negative: LAMP: 62/66 (9WT, 53 MT) positive (94% sensitivity). 15/15 negative (100% specificity) PAN‐mediated LAMP: 9/9 WT negative, 53/53 MT‐positive (100% sensitivity & specificity)
Priya et al. (2022)	*polA* & t*prL*	114 blood samples	DNA extraction (QIAamp DNA Blood Midi Kit)	TPHA	Multiplex High resolution melting (HRM) curve	TPHA identified 56 positive samples, HRM‐LAMP: 54/56 (96%) positive, LAMP: 50/56 positive (89%). All TPHA‐negative were negative in LAMP

Abbreviations: HRM, high‐resolution melting curve; MT, mutant‐type; TPHA, *Treponema pallidum* haemagglutination assay; TPPA, *Treponema pallidum* particle agglutination; WT, wild‐type.

Xiao et al. developed a LAMP assay targeting the *bmp* gene for detecting secondary syphilis (Xiao et al. 2017). The TPPA ( *T. pallidum* Particle Agglutination) assay (an antibody serology test) was used as the reference test. A total of 722 blood samples were analysed, including 624 from suspected secondary syphilis patients and 80 samples from healthy blood donors. The TPPA assay confirmed 603 positive samples; 493 of those were also positive in LAMP (82% sensitivity), while an in‐house PCR could detect only 262 of them (43% sensitivity). Among 129 TPPA‐negative samples, 109 and 111 samples were negative by LAMP and PCR, respectively. No discrepancy analysis was carried out for samples that were only positive in LAMP or PCR. The performance of this assay is difficult to assess since the reference TPPA assay measures antibody and therefore does not indicate the presence or absence of Treponema in a clinical sample.

Tarumoto et al. developed a LAMP assay targeting the 23S rRNA for detecting syphilis along with a peptide nucleic acid (PNA)‐LAMP assay for confirming macrolide resistance (Tarumoto et al. 2020). The PNA clamping probe was designed to be complementary to the wild‐type (WT) sequence. When the relevant sequence of the template DNA is mutated, the PNA probe is unable to bind to the mutant‐type (MT) sequence, leading to a successful LAMP reaction. As reference tests, they chose PCR (targeting the *poIA* gene) and sequencing (for detecting point mutation). A total of 81 skin swabs were tested, including 66 PCR‐positive and 15 PCR‐negative samples; all of the latter were also LAMP negative. Within the 66 PCR‐positive samples, sequencing confirmed 55 and l1 samples as MT and WT, respectively. The LAMP reaction (without PNA) was positive for 62 of PCR‐positive samples (9WT, 53 MT), leading to a 94% agreement rate between the two assays. PNA‐LAMP was negative for all WT samples (100% specificity) and positive for 53 of the mutants (100% sensitivity).

Priya et al. developed a multiplex High‐Resolution Melting curve (HRM) LAMP assay targeting *polA* and *tprL* genes for syphilis diagnosis in blood samples (Priya et al. 2022). The melting characteristics of the nucleic acids were used to differentiate the target amplicons of *polA* and *tprL* genes with Tm values of 80°C and 87°C, respectively. A total of 114 blood samples were analysed, including 64 samples from suspected cases of syphilis infection and 50 samples from seronegative patients. The TPHA ( *T. pallidum* hemagglutination) assay (an antibody serology test) confirmed 56 positive samples; 54 of them were reported to be positive in HRM‐LAMP (96% sensitivity), while conventional LAMP was positive in 50 samples (89% sensitivity). All 56 TPHA‐negative samples were also negative in LAMP.

### 3.5 Herpes Simplex Virus

We identified seven papers for detecting HSV by LAMP (Table 6).

**TABLE 6:** LAMP for detection of herpes simplex virus (HSV).

Study	Target gene	Sample	Sample pre‐treatment	Reference test(s)	Detection mechanism	Results summary
Kaneko et al. (2005)	UL1‐UL2 region for HSV‐1, US4 for HSV‐2	40 genital swabs & 20 ocular smears/fluids	In‐house DNA extraction or directly	PCR‐GE	GE	30 vaginal swabs positive by both LAMP & PCR after DNA extraction. Of those, 21 positive in direct LAMP and only one positive in direct PCR. After DNA extraction, 11 ocular samples tested positive by LAMP, of those 7 positive samples in PCR. Direct LAMP detected 5 out of 11 positive ocular samples; none was positive by direct PCR
Enomoto et al. (2005)	Glycoprotein G (gG)	18 skin lesion swabs samples & 5 genital lesion swab	DNA extraction (NI) or directly	qPCR	GE & turbidity	10 out of 18 skin lesions (in sterile water) were HSV‐1‐positive in LAMP and qPCR either directly or after DNA extraction. All 5 genital swabs (in culture medium) were HSV‐2‐positive by both LAMP and qPCR, only after DNA extraction
Sugiyama et al. (2005)	gG	50 swabs (vulva/cervix)	DNA extraction (QIAamp Blood Kit)	qPCR & virus isolation	Turbidity	LAMP detected 10/12 HSV‐1 (83%) and 14/18 HSV‐2 (78%) pre‐confirmed positive samples in qPCR. Compared to virus isolation, LAMP detected 9/10 HSV‐1 (90%) and 12/12 HSV‐2 (100%) positive samples; one HSV‐1 FP and two HSV‐2 FP for LAMP
Kimura et al. (2005)	gG	69 cerebrospinal fluids	DNA extraction (QIAamp Blood kit)	qPCR & nested PCR	Turbidity	PCR tests: 26 positive (11 HSV‐1 & 15 HSV‐2) and 43 negative; LAMP: 21 (7 HSV‐1 & 14 HSV‐2) positive within 26 PCR‐positive samples (81% sensitivity); 43/43 negative (100% specificity)
Faron et al. (2016)	gG	1153 cutaneous or mucocutaneous swabs	Directly	Enzyme‐linked virus‐inducible system (ELVIS); discrepant samples by AmpliVue	Fluorescence	The LAMP HSV‐1&2 assay by illumigene (Meridian Bioscience) showed sensitivity and specificity of 95% and 95.5%. 73% (57/78) FP and 69% (9/13) FN results in LAMP were supported by the AmpliVue assay
Yang et al. (2016)	gG (only HSV‐2)	73 samples	DNA extraction (E. Z.N.A. R Viral DNA Kit)	PCR	Real‐time turbidity	LAMP in complete agreement with PCR detecting 13 HSV‐2‐positive samples (1 CIN and 12 cervical‐cancer). No HSV‐1 analysis was done. Samples were additionally analysed with HPV LAMP assay
Miyachi et al. (2022)	NI	445 swabs (oral, genital, facial, etc.)	DNA extraction with Eiken Chemical's kit	qPCR	Calcein detection	LAMP with Eiken HSV detection reagent kit Positive agreement rates between qPCR and LAMP were 97% in HSV‐1, and 98% in HSV

Abbreviations: CIN, cervical intraepithelial neoplasms; FN, false‐negative; FP, false‐positive; GE, gel electrophoresis; qPCR, quantitative PCR.

Kaneko et al. developed a LAMP assay to differentiate HSV‐1, HSV‐2 and varicella‐zoster virus by designing three sets of primers against three different genes (Kaneko et al. 2005). A total of 60 clinical specimens were examined, including 40 vaginal swabs (in DMEM) and 20 ocular smears/fluids (aqueous and vitreous fluid) from patients suspected of having genital or ocular herpes, respectively. The authors performed DNA extraction or used the specimen directly for amplification. After DNA extraction of genital swabs (*n* = 40), 30 samples (17 HSV‐1 and 13 HSV‐2) were correctly detected and subtyped by LAMP and PCR, both after DNA extraction. Using direct genital smear suspension (in DMEM), HSV was identified in 70% (21/30) of those samples by LAMP and in just one sample (1/30) by PCR. In ocular fluids after DNA extraction (*n* = 20), eleven and seven samples were HSV‐positive by LAMP and PCR, respectively. No additional test was performed to verify the four discrepant samples, which were regarded by the authors as true‐positives for LAMP. Using direct ocular samples, five of the eleven samples were positive in LAMP, but none were positive in PCR.

Enomoto et al. designed two sets of LAMP primers specific for the glycoprotein G (*gpG*) of HSV 1 and 2 (Enomoto et al. 2005). Turbidity and GE were used as detection mechanisms. A total of 18 swabs were collected (in sterile water) from patients with either gingivostomatitis or vesicular skin eruptions. Of the 18 skin lesions, HSV‐1 could be detected in ten samples (either after DNA extraction or directly) by qPCR, LAMP‐GE, and nine of those could be detected by LAMP‐turbidity. In addition, five genital lesion swabs (*n* = 5) in culture medium were examined, which all were confirmed to be HSV‐2‐positive by qPCR and LAMP (GE/turbidity detection) after DNA extraction. Contrary to skin lesions (in sterile water), the genital swabs (in culture medium) could not be directly detected, which was attributed to the inhibitory effect of culture medium.

Sugiyama et al. developed LAMP assays for HSV subtyping and verified their assay using 50 swabs taken from the vulva and cervix of 25 female patients (Sugiyama et al. 2005). PCR and virus isolation were used as reference tests. Compared to PCR, LAMP detected 83% (10/12) of HSV‐1‐positive and 78% (14/18) of HSV‐2‐positive samples. All PCR‐negative samples were also negative in LAMP. Compared to virus isolation, LAMP detected 90% (9/10) of HSV‐1‐positive and all (12/12) HSV‐2‐positive samples. Within negative isolated samples, three samples (one HSV‐1 and two HSV‐2) were positive in LAMP; the discrepancy was not further resolved. The authors did not disclose the relationship between the PCR and virus isolation results, nor how it was weighted.

In a study by Kimura et al., LAMP assays were developed for detecting and subtyping HSV in cerebrospinal fluids (CSF) (Kimura et al. 2005). The authors analysed 69 CSF samples from 50 patients, including adults and neonates suspected of central nervous system HSV infection. Nested PCR and qPCR were used as reference tests, which both confirmed 26 samples as positive (11 HSV‐1 and 15 HSV‐2) and 43 samples as negative. The clinical sensitivity of LAMP was 81%, detecting 21 samples (7 HSV‐1 and 14 HSV‐2) within 26 PCR‐positive samples. The false‐negative samples (*n* = 5) had viral loads of < 200 copies/mL. All PCR‐negative samples were also negative in LAMP (100% clinical specificity).

The clinical evaluation of FDA‐cleared Illumigene HSV‐1&2 LAMP assay (Meridian Bioscience, Cincinnati, OH) was performed for differentiation of HSV‐1 and HSV‐2 in 1153 cutaneous and mucocutaneous swab specimens (Faron et al. 2016). The enzyme‐linked virus‐inducible system (ELVIS) was used as the reference test. The Illumigene LAMP assay showed overall clinical sensitivity and specificity of 95% and 95.5%. Analysing discrepant samples by Quidel AmpliVue HSV‐1&2 assay (based on HDA isothermal amplification) supported 73% (57/78) of false‐positive and 69% (9/13) of false‐negative LAMP results.

Yang et al. developed an assay for detecting HSV‐2 (and also HPV) in 73 clinical samples, including samples from patients with vaginitis (*n* = 19), chronic cervicitis (*n* = 20), cervical intraepithelial neoplasms (CIN) (*n* = 9) and Level 1 cervical cancer (*n* = 25) samples (Yang et al. 2016). The HSV LAMP data were in complete accordance with the reference test (PCR).

In a work by Miyachi et al., the Eiken LAMP HSV detection reagent kit (Eiken Chemical Co. Ltd., Japan) was used for processing 445 swabs from 211 patients (Miyachi et al. 2022). The swabs were taken from lesions (*n* = 219) and oral mucosa of asymptomatic patients (*n* = 226). The lesion swabs were taken from different anatomical sites including lip, oral mucosa, genital, face, neck, forearm, trunk and lower leg. The qPCR with primers targeting the *gB* envelope gene was used as the reference test, which detected 72 HSV‐1‐positive samples and 41 HSV‐2‐positive samples. All PCR‐positive samples were detected and correctly subtyped by LAMP. Three samples (two HSV‐1 and one HSV‐2 sample) could be additionally identified as positive by LAMP. These samples were considered true positives for LAMP containing low amounts of DNA. All PCR‐negative samples were also negative in LAMP. The reported positive agreement rate between PCR and LAMP was reported to be 97% (72/74) in HSV‐1 and 98% (41/42) in HSV‐2 samples.

### 3.6 Hepatitis B Virus

We identified sixteen publications on HBV‐LAMP assays (Table 7). All studies except one (Cai et al. 2011) used pan‐genotypic primers for HBV detection. The accurate quantitation of hepatitis B viral load using qPCR is the standard management in clinics for screening and monitoring HBV infection. However, only three studies developed quantitative LAMP (qLAMP) assays (Cai et al. 2008, 2011; Lee et al. 2008).

**TABLE 7:** LAMP for detection of hepatitis B virus (HBV).

Study	Target gene	Sample	Sample pre‐treatment	Reference test(s)	Detection mechanism	Results summary
Cai et al. (2008)	Core gene	402 blood specimens	DNA extraction with isolation reagents	qPCR (DaAn Gene Co)	Real‐time fluorescence (quantitative)	295 positive samples confirmed by both qLAMP and qPCR. High correlation between qLAMP and qPCR (*R* ^2^ = 0.96) over the shared dynamic range of the two assays in 254 samples
Lee et al. (2008)	Polymerase	7 serum specimens	DNA extraction (QIAamp Viral DNA MiniKit)	qPCR by Quantiplex HBV DNA Assay	Real‐time turbidity (quantitative)	100% agreement between qLAMP and qPCR; 1 negative & 6 positive samples (viral load of 500 to > 5 × 10^7^ copies/mL)
Cai et al. (2011)	S gene (genotypes B, C)	105 serum samples for quantification & 111 serum samples for genotyping	DNA extraction (QIAamp DNA Blood Mini kit)	qPCR for quantification & PCR sequencing for genotyping	Real‐time fluorescence (quantitative)	High linearity between qPCR and qLAMP viral loads quantification. Of 111 serum samples, LAMP results agreed with PCR in 96% of cases (105/111). Direct sequencing was mostly concordant with PCR results
Nyan et al. (2014)	S gene	182 plasma specimens	DNA extraction (QiAamp DNA Blood Mini Kit) or heat‐spin^a^	Procleix Ultrio qPCR assay	GE	Reference test detected 75 positive samples and 107 negative samples after DNA extraction. Of 75 positive samples, LAMP tested positive for 69 (92%) and 71 (95%) samples after heat‐treatment and DNA extraction, respectively. LAMP showed 100% specificity (107/107)
Akram et al. (2018)	Core gene	200 blood specimens (80 CHB and 120 OBI)	Heat‐spin^a^	qPCR	GE & real‐time fluorescence	PCR detected 50% (40/80) of CHB specimens and 9% (11/120) of OBI specimens. LAMP detected 55% (44/80) of CHB specimens and 36% (43/120) of OBI specimens
Quoc et al. (2018)	S gene	30 blood/plasma samples	Heat‐spin^a^	Real‐time PCR	GE & real‐time fluorescence	PCR after DNA extraction detected 19 positive samples, all were positive in LAMP after heating plasma or blood. The viral loads of samples are not indicated
Pankaew et al. (2019)	S gene	270 plasma specimens	Heat‐spin^a^	qPCR (Abbott)	GE & Turbidity	PCR detected 162 positive and 108 negative samples. LAMP‐turbidity had 90% (146/162) sensitivity and 83% (90/108) specificity. LAMP‐GE had a higher sensitivity (95%, 154/162) but lower specificity (75%, 81/108)
Chen et al. (2019)	pre‐S/S region	127 PCR‐confirmed HBV‐positive plasma specimens with a known copy	125°C, 10 min	Procleix qPCR Ultrio assay	Hand‐ held fluorescence tube scanner, GE & LFD	107 out of 127 samples (84%) tested positive in LAMP. Of 35 samples with ≥ 2500 copies/ml of HBV, 35 (100%) tested positive. Of 37 samples with 500–2500 copies/ml of HBV, 30 (81%) tested positive and 7 (9%) tested negative. For 55 samples with < 500 copies/ml of HBV, 42 (76%) tested positive and 13 (24%) tested negative
Lin et al. (2021)	S gene	82 blood specimens	DNA extraction (PureLink Viral RNA/DNA kit)	ELISA, LFD, qPCR, polymerase spiral reaction (PSR)	GE & direct visual detection of SYBR Green	ELISA, PSR‐LFD, qPCR and LAMP detected 29, 29, 28 and 26 positive samples, respectively. No FP for LAMP. Viral loads of samples are not indicated
Chen, Wang, et al. (2021)	S gene	115 serum samples	Extraction with DNA releasing agent	qPCR (DaAn Gene Co)	LFD	76 samples confirmed as positive by both qPCR (> 30 IU per test) and LAMP. Two samples with 20 IU/test were positive in LAMP‐LFD
Xie et al. (2021)	S gene	20 plasma samples	EX‐DNA/RNA virus extraction kit	Real‐time PCR (Shanghai Kehua Bio‐engineering Co)	Multiplex calcein fluorescence detection in a microfluidic chip	Full agreement between PCR and on‐chip LAMP: 10 positive and 10 negative samples. Viral load of positive samples are not indicated
Vanhomwegen et al. (2021)	Not indicated	950 HBsAg‐positive plasma samples (550 in Senegal & 450 in France)	Heat‐spin^a^ (Senegal) magnetic bead‐based extraction (France)	TaqMan‐based qPCR (ROCHE COBAS)	Calcein detection (Senegal) Real‐time turbidity (France)	LAMP with pan‐genotypic primers led to sensitivity of 98.7% and specificity of 91.5% for diagnosis of DNA ≥ 2 × 10^5^ IU/mL in Senegal. LAMP showed sensitivity of 91% and specificity of 86% for diagnosis of HBV DNA ≥ 2 × 10^4^ IU/mL in France
Ding et al. (2021)	Polymerase	73 serum samples	DNA extraction by nucleic acid releaser	Real‐time PCR	Fluorescence & LFD	PCR detected 32 positive and 41 negative samples LAMP‐Cas12 had 100% sensitivity (32/32) and 100% specificity (41/41)
Maity et al. (2022)	S gene	350 Plasma specimens	DNA extraction (QiAamp DNA Blood MiniKit)	Real‐time PCR (QIAGEN)	Real‐time fluorescence & LFD	PCR detected 150 positive and 200 negative samples. Clinical sensitivity of LAMP‐fluorescence was 95% (143/150) and 92% (138/150) for LAMP‐LFD. No FP in LAMP, 100% specificity. Viral loads of samples are not indicated
He et al. (2023)	S gene	8 Blood samples	Heat‐spin^a^	Real‐Time PCR (Xiamen Amplly Biotechnology)	Colorimetric (hemin/ABTS/H_2_O_2_) or ThT fluorescence	100% agreement between LAMP and PCR; 1 negative & 6 positive samples. This study focused mainly on developing new detection system based on G‐quadruplex formation upon amplification, allowing fluorescence or colorimetric reading
Xu et al. (2024)	S gene	236 plasma samples	DNA extraction by a kit	Real‐time qPCR	Fluorescence	qPCR detected 200 positive and 36 negative samples. LAMP‐Cas 12 showed 99% sensitivity (198/200) and 100% specificity (36/36)

Abbreviations: CHB, chronic hepatitis B; FN, false‐negative; FP, false‐positive; GE, gel electrophoresis; LFD, lateral flow detection; OBI, occult HBV infection; qPCR, quantitative PCR.

^a^ Heat‐spin procedure introduced by Nyan et al. (2014): specimens were diluted with nuclease‐free water and heated at 95°C and 100°C, followed by centrifugation; the supernatant was used for LAMP reaction.

Ting Cai et al. developed a LAMP assay (Cai et al. 2008) for quantification of HBV DNA in serum samples using one set of pan‐genotypic primers targeting the core gene for detecting genotypes B, C and D (no sera positive for subtypes E, F, G or H was available for validation). In this study, 402 pre‐identified serum samples (prior quantified by real‐time TaqMan real‐time PCR (DaAn Gene Co. Ltd., China) with detection range: 10^3^–10^7^ IU/mL) were further reanalyzed by qLAMP. An excellent correlation between qLAMP and qPCR (*R* ^2^ = 0.96) was reported over the shared dynamic range of the two assays in 254 samples.

Zhejun Cai et al. developed qLAMP assays for simultaneous detection and differentiation of genotypes B and C using two sets of genotype‐specific primers (Cai et al. 2011). For quantification, 105 HBV‐positive serum samples were analysed first by qPCR Quantiplex HBV DNA Assay (Chiron Corporation, Emeryville, CA, US) with the quantification range of 10^2^–10^8^ IU/mL. The qLAMP viral loads paralleled well with qPCR, showing a correlation of *R* ^2^ = 0.89 and 0.92 for B‐qLAMP and C‐qLAMP, respectively. For genotyping purposes, serum samples from 111 HBV‐infected patients were tested. LAMP detected 50.5% (56/111) HBV as genotype B, 36% (40/111) as genotype C and 13.5% (15/111) as mixed B/C genotypes. LAMP results agreed with PCR results in 96% (105/111) cases (PCR detected 59 genotype B, 44 genotype C and eight B/C co‐infections). Of six samples determined by LAMP assay to be B/C mixed infections, four were genotype B and two were genotype C by real‐time PCR. Direct sequencing analysis of these samples identified three genotype B, two genotype C and one genotype B/C mixed. Thus, direct sequencing results were 83% (5/6) concordant with results obtained by real‐time PCR.

While quantification is usually completed based on fluorescence, Lee et al. used real‐time turbidity measurement (based on precipitated magnesium pyrophosphate byproduct) to quantify viral loads in seven serum samples (Lee et al. 2008). These samples included six HBV‐positive and one HBV‐negative, as confirmed first by PCR and later by LAMP. Herein, an integrated isothermal device was additionally developed for real‐time monitoring of the turbidity. This device comprises two major components: a disposable polymethyl methacrylate (PMMA) micro‐reactor and a temperature‐regulated optical detection unit. The turbidity after 30 min reaction was linear to initial viral loads. This qLAMP showed high agreement with qPCR and could detect samples with a concentration of < 500 copies/mL.

Most studies purified the blood, serum or plasma samples using commercial kits. Nyan et al. introduced a simple method for pre‐treatment of plasma samples (Nyan et al. 2014). In this method, specimens were first diluted with an equal amount of deionised distilled water, followed by incubation at 95°C for 5 min and 100°C for 5 min, and further collecting DNA from the supernatant after centrifugation at 12,000 *g* for 5 min. A total of 182 plasma samples were analysed. The reference Procleix Ultrio PCR assay (Gen‐Probe, Emeryville, California) detected 75 positive samples, while LAMP detected 69 samples of them (92% sensitivity) after heat treatment. LAMP detected two additional samples (71/75, 95% sensitivity) when extracted DNA was used. This pre‐treatment technique was further applied for plasma pre‐treatment by other groups (Akram et al. 2018; Quoc et al. 2018; Pankaew et al. 2019; Vanhomwegen et al. 2021; He et al. 2023), although the heating duration and settings of the centrifugation step were slightly varied among these studies.

Akram et al. included 80 CHB (chronic hepatitis B) and 120 potential OBI (occult HBV infection) patients (Akram et al. 2018). Among the CHB sample, 50% (40/80) were positive by qPCR. The HBV‐DNA levels detected by qPCR ranged between 10^2^–10^4^ IU/mL in 20% (16/80) and > 10^5^ IU/mL in 30% (24/80) of samples. LAMP identified 55% (44/80) of CHB samples as positive, including those detected by qPCR. qPCR detected 9% (11/120) of potential OBI cases (viral loads of 10^2^–10^5^ IU/mL), whereas LAMP identified HBV‐DNA in 36% (43/120) of samples, including those detected by qPCR. The authors considered discordant samples (*n* = 32) as true positives for LAMP, without conducting a discrepancy analysis by an independent assay.

Pankaew et al. verified their LAMP assay (Pankaew et al. 2019) on 270 plasma specimens consisting of 162 significant (> 2 × 10^5^ IU/mL) and 108 non‐significant (< 2 × 10^5^ IU/mL) viremia according to qPCR results. The authors reported 90% (146/162) sensitivity and 83% (90/108) specificity for LAMP‐turbidity. The GE analysis of LAMP products had a relatively higher sensitivity (95%, 154/162), but lower specificity (75%, 81/108).

Chen et al. used heating at 125°C for 10 min for plasma pre‐treatment and verified their LAMP assay on 127 PCR‐confirmed HBV‐positive plasma specimens with a known copy number (Chen et al. 2019). Among 35 samples that had greater than 2500 copies/ml of HBV‐DNA, 35 (100%) tested positive using LAMP. Of 37 samples with 500–2500 copies/ml, 30 (81%) tested positive and 7 (9%) tested negative in LAMP. For 55 samples that had < 500 copies/ml of HBV‐DNA, 42 (76%) tested positive and 13 (24%) negative using LAMP. Overall, 84% of samples (107/127) verified positive in LAMP using only heated plasma samples.

A self‐driven microfluidic chip was developed (Xie et al. 2021), which is composed of multiple reaction chambers, fluidic channels, and a vacuum chamber (to speed‐up sample loading). By pre‐setting LAMP primers specific to different targets in different reaction chambers, the chip can theoretically be used for multiplex detection of STIs and other pathogens. The chip can be read out by a smartphone camera via fluorescence in which positive reactions appeared as bright fluorescence dots. The focus of this work has been on the validation of the platform, and only 20 plasma samples were analysed, which showed consistent data with real‐time PCR.

Vanhomwegn et al. designed a set of degenerate pan‐genotypic LAMP primers specific to eight major HBV genotypes/sub‐genotypes (A1/2/3/B/C/D/E/F) (Vanhomwegen et al. 2021). The Roche Cobas TaqMan real‐time PCR was used as the gold standard. A total of 550 seropositive samples were analysed in Senegal using the heat spin pre‐treatment method as described by (Nyan et al. 2014). The LAMP results were visually detected, which showed a sensitivity of 99% and a specificity of 91.5% for the diagnosis of HBV DNA ≥ 2 × 10^5^ IU/mL. In addition, 450 seropositive samples were analysed in France after magnetic bead‐based extraction and further detected by real‐time turbidity, which led to a sensitivity of 91% and a specificity of 86% for the diagnosis of HBV DNA ≥ 2 × 10^4^ IU/mL. The limitation of this study is that their assay is not adapted to discriminate low‐level viraemia.

Two studies combined LAMP with clustered regularly interspaced short palindromic repeats (CRIPSR)‐associated (Cas) method to increase the sensitivity of detection (Ding et al. 2021; Xu et al. 2024). In this technique, the HBV DNA is first pre‐amplified by LAMP to generate more target sequence substrates for the guide RNA (gRNA)/Cas12 system. The sequence of gRNA should be designed carefully for specific recognition of the DNA target. Detection of the amplified DNA by gRNA/Cas12 triggers the collateral cleavage of Cas12, which cleaves a single‐stranded DNA (ssDNA) reporter molecule. The cleaved ssDNA can then be visualised by fluorescence readout or LFD. Ding et al. (Ding et al. 2021) verified their assay on extracted DNA from 73 plasma specimens. Both real‐time PCR and CRISPR‐assisted LAMP detected 32 positive samples and 41 negative samples, leading to 100% clinical sensitivity and specificity for LAMP. In the recent work by Haipo Xu et al. (Xu et al. 2024), a one‐pot method based on CRISPR/Cas12‐assisted LAMP was developed to detect A‐F genotypes of HBV. The assay was tested on extracted DNA from 236 plasma samples. The real‐time qPCR identified 200 samples as positive and 36 samples as negative. The detection performance of this assay is a sensitivity of 99% (198/200) and specificity of 100% (36/36), respectively.

### 3.7 Human Immunodeficiency Virus‐1

For HIV‐1 diagnosis by LAMP, we identified 13 publications (Table 8). As HIV is a single‐stranded RNA retrovirus, amplification needs a reverse transcriptase (RT) step to convert RNA to DNA to allow amplification of both RNA and proviral HIV DNA.

**TABLE 8:** Reverse transcriptase (RT)‐LAMP for detection of human immunodeficiency virus‐1 (HIV‐1).

Study	Target gene	Sample	Sample pre‐treatment	Reference test(s)	Detection mechanism	Results summary
Curtis et al. (2008)	Protease & p24	5 plasma and 5 whole‐blood samples from seropositive donors	Diluting/heating or RNA extraction (Viral RNA mini kit)	HIV RNA bDNA (Chiron, Everyville) for plasma & Roche COBAS Amplicor for blood	GE & PicoGreen fluorescence detection under UV	Reference tests positive for all samples. LAMP detected 4 out of 5 plasma samples and 3 out of 5 blood samples after RNA extraction. 3 out of 5 heat‐treated plasma and 4 out of 5 heat‐treated bloods were detected
Curtis et al. (2009)	Protease & p24	9 whole‐blood samples from 8 seropositive patients and 1 seronegative donor	Dilution 1:4 with a red blood cell lysis buffer	PCR HIV RNA bDNA (Chiron, Everyville)	GE & PicoGreen fluorescence detection under UV	All samples correctly identified by LAMP and PCR
Hosaka et al. (2009)	Integrase (group M)	97 plasma specimens	RNA extraction with guanidine thiocyanate	Genotyping, Abbott HIV‐Ab test, RT‐PCR	GE, turbidity & visual detection of fluorescence under UV	Of 57 samples from infected individuals, 56 harboured group‐M HIV‐1 strains, which were all positive in RT‐LAMP (100% sensitivity); one sample (1/57) harbouring group‐O and 40 HIV‐1‐uninfected samples were RT‐LAMP‐negative
Zhao et al. (2012)	Integrase	173 plasma samples	RNA extraction (TaKaRa kit)	WB, ELISA, RT‐PCR	GE, visual detection of SYBER Green fluorescence	18 WB‐confirmed positive samples: 18/18 positive in LAMP, 17/18 positive in PCR; 153 negative samples from healthy donors as confirmed by ELISA: 153/153 negative by LAMP, 153/155 negative in PCR, 2 FP in PCR
Zeng et al. (2014)	p24	52 seropositive plasma samples	RNA extraction (QIAamp Viral RNA Mini Kit)	RT‐qPCR (Qiagen)	Real‐time fluorescence (quantitative)	4 samples negative in both PCR and LAMP, 6 samples with viral loads outside quantification limit of LAMP. Within 42 qualified samples, qLAMP had dynamic range of 2.5 × 10^2^ to 10^7^ copies, Pearson correlation analysis with PCR (*R* ^2^ = 0.859)
Rudolph et al. (2015)	RT or integrase	Serial plasma specimens from 12 donors	RNA extraction (QIAamp Viral RNA Kit)	RT‐PCR & four immune tests	Fluorescence detection under UV	RT‐LAMP detected seroconverting individuals 1–3 weeks earlier than the immune assay, but not as sensitive as the RT‐PCR APTIMA assay
Odari et al. (2015)	Integrase (group M)	364 plasma samples	Viral extraction (Roche High Pure Viral Nucleic Acid Kit)	RT‐qPCR assay (Abbott)	Real‐time fluorescence	RT‐qPCR: 231 samples as positive, 115 samples as negative. RT‐LAMP showed overall sensitivity of 88% (203/231) and specificity of 99% (114/115)
Gurrala et al. (2016)	Integrase	991 plasma samples (164 of those tested on the chip)	RNA extraction (Qiagen Viral RNA Minikit)	RT‐qPCR (Versant)	pH sensitive	In tube LAMP: detection rate of 95% (465/489) for viral loads (VL) > 1k copies (per reaction), 89% (284/320) for VL 50–1k copies, 41% (75/182) for VL < 50 copies. On‐chip LAMP: detection rate of 89% (31/35) for VL > 1k copies, 76% (48/63) for VL 50–1k copies, 21% (14/66) for VL < 50 copies
Curtis et al. (2016)	RT (subtype B)	20 plasma samples	RNA extraction (QIAamp Viral RNA Mini Kit)	RT‐qPCR assay (Abbott)	Fluorescence detection under UV	PCR detected 15 samples as positive and 5 as negative. LAMP in NINA^a^ heater detected 9 samples with viral loads (VL) 10^4^ copies/mL; 4 samples with VL < 10^4^ copies/mL. Two samples with VL < 2000 copies/mL were not detected. PCR‐negative samples were all negative in LAMP
Curtis et al. (2018)	Integrase (group M)	61 plasma samples	RNA extraction (QIAamp Viral RNA Mini Kit)	Real‐time qPCR by roche COBAS AmpliPrep/COBAS TaqMan HIV	Real‐time fluorescence	RT‐qPCR confirmed 57 positive samples, 45 of them were also positive in LAMP. Four RT‐PCR‐negative samples were positive in LAMP
Li et al. (2021)	Integrase	127 plasma samples	RNA extraction (Roche HighPure Viral RNA Kit)	ELISA, WB, PCR‐sequencing, RT‐qPCR	LFD, colorimetric & real‐time fluorescence	ELISA/WB detected 93 positive samples. Of those, RT‐qPCR and variant‐resistant RT‐LAMP, detected 78 and 71 positive samples, respectively. Within 39 positive samples by variant‐resistant RT‐LAMP, conventional RT‐LAMP detected 21 of them
Zhang, Li, et al. (2022)	Integrase	101 seropositive plasma samples	RNA extraction (Roche High Pure Viral RNA kit)	RT‐qPCR (DaAn Gene Co)	Real‐time fluorescence	Of 98 RT‐qPCR‐positive samples, 88 were positive in variant‐resistant RT‐LAMP (89% sensitivity). No FP in LAMP (100% specificity)
Khan et al. (2023)	Integrase	25 seropositive blood samples	Nucleic acid extraction form plasma, heat‐treated plasma, lysis buffer/heat‐treated whole blood and DBS	RT‐qPCR	HNB colour, GE & fluorescence	15 positive samples in qPCR; LAMP was positive in 16 samples for the first two purification methods, 17 for the other two methods, no discrepancy analysis

Abbreviations: DBS, lysis buffer‐treated dried blood spot; FN, false‐negative; FP, false‐positive; GE, gel electrophoresis; LFD, lateral flow detection; qPCR, quantitative PCR; RT, reverse transcriptase; WB, Western blotting.

^a^ NINA (non‐instrumented nucleic acid amplification) heating device generates heat from the exothermic reaction of calcium oxide and water.

Curtis et al. developed a RT‐LAMP assay and verified its performance on 10 samples from HIV‐1‐positive patients (five plasma and five whole blood), using either extracted RNA or heated clinical samples (Curtis et al. 2008). For the latter, plasma and blood samples were first diluted in RNase free water to prevent coagulation upon heating, and then heated. Using RNA extracted samples, they could detect four out of the five plasma samples and three out of the five blood samples. On the other hand, they could detect three out of five heat‐treated plasma and four out of five heat‐treated blood samples. In another work, the same authors (Curtis et al. 2009) assessed this assay to directly detect HIV‐1 in whole blood samples by diluting (1:4) with a red blood cell lysis buffer. Whole‐blood samples from eight different HIV‐1‐infected patients and one HIV‐1 seronegative donor were tested and could be detected correctly by LAMP. In another study (Khan et al. 2023), differently pre‐treated samples were applied for RT‐LAMP including nucleic acid extracted from plasma, heat‐treated plasma, lysis buffer/heat‐treated whole blood and lysis buffer‐treated dried blood spot (DBS). Within 21 seropositive samples, RT‐qPCR detected 15 positive samples, while LAMP detected one additional positive sample (*n* = 16) using nucleic acid extracted or heat‐treated plasma and two additional positive samples (*n* = 17) if using lysis buffer/heated blood or DBS. No discrepancy analysis was performed for samples (*n* = 3), which were positive only in LAMP. The limitation of all the above‐mentioned studies is the small sample sizes.

Hosaka et al. developed a RT‐LAMP assay for detection of group M HIV‐1 in plasma specimens (Hosaka et al. 2009). Of 57 samples from infected individuals, 56 harboured group‐M HIV‐1 strains (such as subtypes A, B, G, F2, and circulating recombinant forms (CRFs)), which all were positive in RT‐LAMP. Uninfected samples (*n* = 40) and one sample harbouring group‐O HIV‐1 were negative by RT‐LAMP. Curtis et al. additionally developed a group M HIV‐1 RT‐LAMP (Curtis et al. 2018) and further verified the assay on 61 plasma samples with considerable subtype diversity, encompassing subtypes A1, C, D, G, CRF02_AG, and CRF11_cpx. The RT‐qRCR (Roche) was used as the reference test and to quantify viral loads, which confirmed 57 samples as positive. RT‐LAMP detected 94% (33/35) of the specimens with viral loads of ≥ 10^4^ RNA copies/mL and 86.5% (45/52) of specimens with viral loads of ≥ 10^3^ RNA copies/mL. Within five RT‐PCR‐positive specimens with viral loads of < 10^3^ RNA copies/mL, only one was positive in RT‐LAMP. All RT‐PCR‐negative samples (*n* = 4) were positive by RT‐LAMP. No further analysis was conducted to resolve these discrepant samples.

Zeng et al. developed a RT‐LAMP for the quantitative detection of HIV‐1 (Zeng et al. 2014). The linear correlation between RT‐LAMP and RT‐PCR was significant by both Pearson correlation analysis (*R* ^2^ = 0.859) and regression analysis (*R* ^2^ = 0.737) in 42 samples with detectable viral loads. Odari et al. (2015) also developed a RT‐LAMP assay as a semi‐quantitative procedure to measure HIV‐1 group M viral load and verified their assay on 346 samples from patients in Kenya and Germany. As the reference test, the RT‐qPCR test (Abbott) was used, which confirmed 231 samples as positive (viral loads > 5000 copies/mL) and 115 samples as negative. RT‐LAMP showed an overall clinical sensitivity of 88% (203/231) and specificity of 99% (114/115). The authors considered their results not sufficient to allow quantitative measurement of viral load as opposed to the study by Zeng et al. (2014). Odari et al. (2015) attributed this to the fact that in the latter study, only subtypes specific to the China region were used, whereas they analysed HIV‐1 group M subtypes circulating globally (Kenya and Germany), and that they analysed 231 positive samples, while Zeng et al. analysed only 42 samples.

Rudolf et al. assessed the utility of a RT‐LAMP assay for detection of acute HIV‐1 infection by directing primer sets against highly conserved regions within the RT or INT (integrase) gene (Rudolph et al. 2015). Serial plasma specimens were collected from 12 donors who became HIV‐1 infected during the collection period. A total of five commercial tests were included as reference tests, including four immunological tests from Bio‐Rad (Multi‐spot HIV‐1/HIV‐2 Rapid Test, GS HIV‐1 Western blot, third‐generation GS HIV‐1/HIV‐2 Plus O EIA and fourth‐generation GS HIV ComboAg/Ab EIA) and the FDA‐approved Hologic APTIMA HIV‐1 RT‐PCR Assay. The RT‐LAMP detected seroconverting individuals 1–3 weeks earlier than the Multi‐spot HIV‐1/HIV‐2 rapid antibody test and up to 2 weeks earlier than the fourth‐generation antigen/antibody (Ag/Ab) combo enzyme immunoassay (EIA), which is the most sensitive among the four tested immune assays. However, RT‐LAMP was not as sensitive as the RT‐PCR APTIMA assay, which the authors attributed to the difference in the input sample volume between the two assays (as 7% of the sample required for APTIMA PCR assay was used for LAMP). No significant difference was observed between the functionality of the RT and INT primers.

In another work, a metal oxide semiconductor (CMOS) microfluidic chip was developed to simultaneously amplify and detect HIV‐1 RNA (Gurrala et al. 2016). The CMOS chip is pH sensitive; when pH changes, the ISFET (ion sensitive field effect transistor) generates an electrical signal. After loading the extracted RNA on the chip, it is inserted into the chip holder and the reaction is powered via a standard USB port on a PC. Bespoke software on the PC then converts the pH data received by the ISFETs to filtered digital information, which could be visualised real‐time on the screen via graphic user interface in the form of a plot of the mV of signal produced and the time of reaction in seconds. The authors first analysed 991 clinical samples with LAMP in a reaction tube, which led to the detection rate of 95% (465/489) for inputs > 1000 copies/reaction, 89% (284/320) for inputs of 50–1000 copies and 41% (75/182) for inputs < 50 copies/reaction. Afterwards, they analysed 164 clinical plasma specimens with the CMOS chip. The proportion of positive reactions was 89% (31/35) for inputs > 1000 copies/reaction, 76% (48/63) for 50–1000 copies/reaction and 21% (14/66) for < 50 copies/reaction.

HIV‐1 has very high sequence diversity. The mismatches between primers (especially in the 3′‐end) and templates significantly reduce the efficiency of amplification. To tackle this issue, one approach is to target two separate regions within the HIV genome to circumvent mismatches and facilitate amplification. This approach has been used in several commercial tests such as Hologic APTIMA HIV‐1 PCR assay or Roche COBAS Taq‐Man HIV‐1 test. As an alternative mitigation strategy, Li et al. developed a variant‐resistant RT‐LAMP method for detecting various genotypes (subtypes/CRFs/URFs) of the HIV‐1 M group (Li et al. 2021). This novel method features the addition of as little as 0.15 U of high‐fidelity DNA polymerase to the standard (25 μL) LAMP reaction mixture, to remove the mismatched bases occurring at the 3′‐end of their LAMP primers during amplification. Two batches of plasma samples were analysed in which the HIV‐1 infection status was prior identified by ELISA or Western blot (WB). The first batch consists of 82 plasma samples including 60 positive and 22 negative for HIV‐1. Within pre‐confirmed positive samples (*n* = 60), RT‐qPCR, variant‐resistant RT‐LAMP, and conventional RT‐LAMP assays detected 45, 39 and 21 HIV‐1‐positive, respectively. Thus, the novel variant‐resistant RT‐LAMP assay detected 18 positive samples that were missed by the conventional RT‐LAMP, confirming that the new format is more suitable for detecting high‐variation viruses. All six positive samples that failed to be detected by the novel RT‐LAMP assay showed high Ct values of > 38 in the RT‐qPCR analysis, implying a very low copy number. The second batch consisted of 45 plasma samples including 33 positive and 12 negative samples for HIV‐1. Among pre‐confirmed 33 positive samples, 33 and 32 were positive in RT‐qPCR and resistant‐tolerant RT‐LAMP, respectively. Another study used a similar approach and implemented a high‐fidelity DNA polymerase in their RT‐LAMP assay to detect various HIV‐1 genotypes and unique recombinants including CRF01_AE, CRF07_BC, CRF08_BC, CRF55_01B and URFs (Zhang, Li, et al. 2022). The assay was verified with 101 seropositive plasma samples. Within 98 RT‐qPCR‐positive samples, RT‐LAMP detected 87 of them (89% sensitivity). The failure of RT‐LAMP in detecting 11 PCR‐positive samples was attributed to the lower sample volume input in the LAMP assay and degradation of samples after multiple free thaw cycles.

## 4 Discussion

The clinical outcomes of multiple studies reviewed here show that LAMP can be as sensitive and specific as PCR. In these studies, LAMP results were compared with one or more reference tests (usually PCR‐based assays). Several of these studies included an independent assay to resolve discrepancies between LAMP and the reference test. However, not all studies resolved discrepancies and considered their results as true‐positive or true‐negative, compromising the ability to measure the true clinical sensitivity and specificity of their developed LAMP assay. Comparing the performance of these reported assays is challenging due to variability in extraction methods, amplification protocols, detection systems and numbers of tested samples. Each of these parameters significantly affects sensitivity, specificity, accuracy and reproducibility of the assay.

One of the major advantages of LAMP is the possibility to use versatile readout methods, including turbidity, calcein detection, real‐time or end‐point fluorescence, colorimetric and LFD. Colorimetric is suitable for PoC purposes; however, colorimetric detection, particularly those based on pH alteration, is reliable only if the nucleic acid template is relatively pure and in a solution with neutral pH. However, clinical specimens and even those that undergo partial purification, usually contain chemicals that may significantly affect pH. On the other hand, the colorimetric readout based on the formation of magnesium pyrophosphate using the HNB dye leads to a subtle colour change (violet to blue) and does not adequately differentiate between positive and negative samples, leading to indeterminate results (Choopara et al. 2017). The application of metal (Zn^2+^ or Mn^2+^) indicators, namely pyridylazophenol dyes (PAPS), has been recently demonstrated for the metallochromic detection of LAMP products (Zhang, Hunt, et al. 2022). This technique is pH independent and leads to a discernible colour change. Recently, the bromo‐PAPS/Zn^2+^ complex has been successfully used for the colorimetric detection of SARS‐Covid‐2 in gargle samples after a rapid simple inactivation procedure (Zhang, Hunt, et al. 2022; Szobi et al. 2023). The potential of PAPS dyes for detecting variant clinical specimens directly or after a minimal treatment step is yet to be explored. LFD also allows a reliable and affordable readout; though in all surveyed papers in this review, the reaction vial had been opened to transfer amplified products to the LFD strip, which causes amplicon contamination and false‐positive results. A mitigation strategy is to use an enclosed device in which opening the amplification vial and the LFD readout occurs in a closed space. An example of such a device is the disposable cassette from Quidel (San Diego, CA) (Gaydos et al. 2016). A limitation of LAMP is believed to be the higher risk of amplicon contamination and thus more false‐positives than other NAATs. This, however, occurs mainly due to the lack of familiarity with the method and is avoidable by following good laboratory practice principles and by implementing closed systems for amplification and detection.

As is evident in Tables 2, 3, 4, 5, 6, 7, 8, most studies performed nucleic acid extraction using commercial kits or in‐house methods, which rely on trained personnel for processing, require reagents and may involve multiple heating and centrifugation steps. Several studies used simple pre‐treatment approaches such as crude cell lysis by nucleic acid releasing agents or applied the combination of centrifugation with lysis and heating. Only a few studies evaluate using specimens directly or after a short heating step. While the *Bst* polymerase in LAMP is reported to be rather tolerant to amplification inhibitors, clinical specimens, particularly blood or urine, usually require pre‐treatment. Besides, depending on the specimen type and the pathogen load, a concentration step is often necessary to increase sensitivity. A major bottleneck for the widespread application of generally any isothermal assay, including LAMP, is believed to be the need for nucleic acid extraction, particularly from complex clinical specimens (Everitt et al. 2021). Using simple and quick purification techniques is therefore crucial to make LAMP and other NAATs suitable for point‐of‐care purposes. An example of such nucleic acid purification is the ultrarapid (under 30s) and affordable dipstick DNA extraction method (Zou et al. 2017; Mason and Botella 2020). Using this technique, nucleic acids could be purified from clinical samples by dipping cellulose‐based dipsticks into three solutions: (1) the nucleic acid extraction buffer containing the specimen; (2) the wash buffer to remove impurities; and (3) the amplification reaction to elute the nucleic acids. This method is designed for speed and simplicity, so the yield of extracted nucleic acid is less than commonly used solid‐phase nucleic acid extraction techniques. Depending on the specimen type, such purification techniques could lower the sensitivity but would make the assay more compliant with the WHO ASSURED criteria by ensuring affordability, user‐friendliness and rapidity (Peeling et al. 2006). For example, the Eiken chemical LAMP assay (Eiken Chemical Co Ltd., Japan) for detecting *Mycobacterium tuberculosis* applies a simple, quick and inexpensive cartridge‐based method for extracting DNA from sputum. Despite having significantly less clinical sensitivity compared to the Cepheid Xpert PCR assay (45.5% vs. 87.8%), this LAMP assay had been endorsed by WHO in 2016 (Eddabra and Ait Benhassou 2018).

Although co‐infection with STIs is common (Chung et al. 2023), only very few studies reviewed here addressed this (Xu et al. 2016; Yang et al. 2016; Chen et al. 2023). Compared to PCR, achieving the simultaneous detection of several targets in a single tube (multiplexing) is more challenging with LAMP. However, multiplexing for detecting two or three targets can be achieved relatively easy. For detailed review on LAMP multiplexing, the reader is referred to Crego‐Vicente et al. (2024).

In summary, LAMP assays hold great promise to be used for detection of STIs at the PoC due to its isothermal nature, rapid amplification time, low cost, easy set‐up, robustness, high sensitivity, excellent specificity and versatile detection. Despite advantages and recent advancements, broad adoption of LAMP remains limited. A recent market overview that comprehensively analysed a dataset of 1134 LAMP patent documents and 21 LAMP‐related clinical trials, however, suggests an upcoming and promising future for widespread use of LAMP for rapid testing of pathogens in resource‐limited settings (Feddema et al. 2024). Although an all‐in‐one PoC prototype including a simple sample pre‐treatment, isothermal amplification of multi‐pathogens with a reliable readout, suitable for high‐burden and resource‐limited settings remains elusive, with funding and political commitment this technology could be developed and made available to those areas of the world that need it most.

## Author Contributions

**Yasaman Ahmadi:** writing – review and editing, writing – original draft, visualization, investigation, methodology. **Yejiong Yu:** methodology, writing – review and editing. **Zhanfeng Cui:** supervision, funding acquisition, resources. **Wei E. Huang:** supervision, writing – review and editing, funding acquisition, conceptualization, resources, investigation. **Monique I. Andersson:** resources, supervision, funding acquisition, writing – review and editing, investigation, conceptualization.

## Conflicts of Interest

The authors declare no conflicts of interest.

## Data Availability

The authors have nothing to report.
